# Simultaneous Structural Monitoring over Optical Ground Wire and Optical Phase Conductor via Chirped-Pulse Phase-Sensitive Optical Time-Domain Reflectometry

**DOI:** 10.3390/s24227388

**Published:** 2024-11-20

**Authors:** Jorge Canudo, Pascual Sevillano, Andrea Iranzo, Sacha Kwik, Javier Preciado-Garbayo, Jesús Subías

**Affiliations:** 1 Department of Applied Physics, Universidad de Zaragoza, 50009 Zaragoza, Spain; psevi@unizar.es (P.S.); jesus.subias@unizar.es (J.S.); 2 Aragón Photonics Labs, 50009 Zaragoza, Spain; a.iranzo@aragonphotonics.com (A.I.); j.preciado@aragonphotonics.com (J.P.-G.); 3 Red Eléctrica de España, 28109 Alcobendas, Spain

**Keywords:** distributed acoustic sensing, Rayleigh scattering, phase-sensitive OTDR, sag measurement, overhead line monitoring

## Abstract

Optimizing the use of existing high-voltage transmission lines demands real-time condition monitoring to ensure structural integrity and continuous service. Operating these lines at the current capacity is limited by safety margins based on worst-case weather scenarios, as exceeding these margins risks bringing conductors dangerously close to the ground. The integration of optical fibers within modern transmission lines enables the use of Distributed Fiber Optic Sensing (DFOS) technology, with Chirped-Pulse Phase-Sensitive Optical Time-Domain Reflectometry (CP-ΦOTDR) proving especially effective for this purpose. CP-ΦOTDR measures wind-induced vibrations along the conductor, allowing for an analysis of frequency-domain vibration modes that correlate with conductor length and sag across spans. This monitoring system, capable of covering distances up to 40 km from a single endpoint, enables dynamic capacity adjustments for optimized line efficiency. Beyond sag monitoring, CP-ΦOTDR provides robust detection of external threats, including environmental interference and mechanical intrusions, which could compromise cable stability. By simultaneously monitoring the Optical Phase Conductor (OPPC) and Optical Ground Wire (OPGW), this study offers the first comprehensive, real-time evaluation of both structural integrity and potential external aggressions on overhead transmission lines. The findings demonstrate that high-frequency data offer valuable insights for classifying mechanical intrusions and environmental interferences based on spectral content, while low-frequency data reveal the diurnal temperature-induced sag evolution, with distinct amplitude responses for each cable. These results affirm CP-ΦOTDR’s unique capacity to enhance line safety, operational efficiency, and proactive maintenance by delivering precise, real-time assessments of both structural integrity and external threats.

## 1. Introduction

Due to the rise in multiple energy sources and the escalating demand, conventional electrical grids are undergoing a shift toward intelligent grid systems. The implementation of intelligent grid systems demands robust monitoring and control of power transmission infrastructure, particularly overhead lines. By continuously monitoring the health and performance of these lines, utilities can proactively identify potential issues, optimize maintenance schedules, and enhance system reliability. Real-time monitoring of overhead lines provides valuable insights into factors such as mechanical stress, environmental impacts, and security threats. This information enables timely intervention, preventing costly outages and ensuring the uninterrupted supply of electricity to consumers. Moreover, the data collected from these monitoring systems can be integrated into advanced analytics and machine learning algorithms to optimize grid operations and facilitate the integration of renewable energy sources [1].

Distributed Acoustic Sensing (DAS) technology offers a revolutionary approach to structural monitoring of overhead lines [2]. By leveraging the existing fiber optic cables within these lines, DAS enables continuous, real-time monitoring of the entire infrastructure. This technology can detect a wide range of events, including mechanical vibrations, impacts, and environmental factors [3], providing early warning of potential issues, such as cable damage, tower instability, or unauthorized access. Additionally, DAS systems can be used to optimize maintenance schedules, reduce downtime, and improve overall system reliability. By integrating DAS with other smart grid technologies, utilities can achieve a more comprehensive and proactive approach to managing their overhead line assets.

Throughout the transmission process, it is crucial to consistently uphold balance in order to align power supply with demand [4]. Global climate changes exert an influence on the performance of our current electrical transmission lines. To prevent overloads, phase imbalances, and fluctuations, the electric current within the lines must be carefully controlled. Additionally, monitoring the position of the line is essential for addressing sagging and galloping occurrences, as sagging can bring the conductor dangerously close to the ground, posing safety and operational risks [5]. Nowadays, many overhead installations reduce their conduction capacity based on worst-case weather scenarios to avoid exceeding the sag limits, which greatly reduces their efficiency [6]. The real-time monitoring of sag provides the capability for dynamic adjustments to ampacity, ensuring a fine-tuned balance between operational efficiency and security. By continuously assessing and responding to sag in the transmission lines, it becomes possible to optimize the current-carrying capacity without compromising the overall security and reliability of the system. This proactive approach not only safeguards against potential issues like overloads but also contributes significantly to the efficiency and performance of the entire power transmission line.

Methods for estimating current sag often rely on establishing correlations between this parameter and various physical attributes of the transmission line, such as temperature, tension, or other factors [7]. However, these approaches typically involve the installation of individual sensors, a solution that can be both costly and susceptible to failures [8]. In the contemporary installation of overhead lines, the incorporation of power transmission and communication features is a common practice, accomplished by integrating one or more conductors that house deployed optical fibers within the structure of the line [9]. The Optical Phase Conductor (OPPC) has emerged as the preferred choice for new overhead line installations due to its ability to address several challenges encountered by alternative technologies, like Optical Ground Wire (OPGW) or All Dielectric Self Supported (ADSS). Furthermore, the adoption of an OPPC brings additional benefits, particularly in scenarios where the upgrading of older overhead lines is required. In such cases, where towers may not be equipped to accommodate additional wires, an OPPC provides a seamless solution. Its incorporation does not necessitate extra installations or redesigns, making it a practical and cost-effective choice for modernizing existing infrastructure [10]. The embedded optical fibers serve a dual purpose by facilitating both power transmission and communication functions within a single conductor. This not only streamlines the overall design but also enhances the adaptability and efficiency of the power transmission system.

In the present study, the previously developed technique of sag monitoring using distributed optical fiber sensing in the OPGW [11] is, to the best of our knowledge, extended for the first time to the actual electrical conductor of the overhead installation, the OPPC. This advanced methodology involves the deployment of an interrogator unit designed to measure the strain experienced by the optical fiber within the cables, capturing and recording its dynamic variations over time. The obtained strain data are then subjected to a transformation into the frequency domain, enabling a profound analysis of cable vibrations induced by external factors. The employed Chirped-Pulse DAS technology enables high-fidelity frequency analysis by providing quantitative, linear measurements with a constant SNR throughout the monitored distance. The measured data can then be divided into the high-frequency domain (above 20 Hz), where mechanical interventions and intrusions can be detected [12], or the low-frequency domain (below 2 Hz), where wind-induced vibrational data can be retrieved for sag monitoring purposes [13]. By closely tracking changes in the vibration frequencies of both lines, sag can be estimated for the OPPC while using the information from the OPGW to distinguish between environmental temperature contributions and elongation due to the heat derived from electrical conduction in the OPPC. This method provides a direct and real-time assessment of sag alterations over the course of time, with clear advantages over traditional methodologies. This technique not only enhances reliability but also proves to be a more cost-effective solution when compared to existing technologies currently in use. This in-depth analysis yields valuable insights into the dynamic behavior of the cable, facilitating the derivation of quantitative instantaneous sag values for each span. Such a detailed understanding of the vibrational characteristics enhances the precision and accuracy of sag monitoring, contributing to the overall effectiveness of the proposed CP-ΦOTDR technique in the realm of overhead line analysis and management.

## 2. Measurement Principle

Optical Time-Domain Reflectometry (OTDR) presents distinct advantages for extensive infrastructure monitoring when compared to electronic alternatives. This technology allows for distributed sensing across expansive distances at a reduced cost, leveraging the optical dual role of the optical fiber both as a sensor and information carrier. This dual functionality minimizes the need for multiple measurement devices and enhances resilience to electromagnetic interference [14,15,16,17]. When employing a highly coherent pulsed laser as a light source, the interference of Rayleigh backscattered light, occurring as the optical pulse interacts with scattering centers, produces a characteristic jagged profile. Minute perturbations in the fiber induce local changes in the refractive index, leading to random amplitude variations in the recorded signal [18,19,20]. This technique, commonly known as Phase-Sensitive OTDR (ΦOTDR), identifies and locates external perturbations near the fiber with high sensitivity by analyzing the variation in backscattered intensity traces between pulses. One of the main disadvantages of this technology is the existing nonlinear relationship between the variation in the retrieved signals and the amplitude of the original perturbation [21]. A deeper analysis on the phase of the signal through I/Q demodulation and heterodyne detection can provide a quantitative measurement of the amplitude, partially solving this problem [22]. However, the 2π periodicity of the phase and the presence of fading points are inherent to the architecture and limit its overall performance.

Chirped-Pulse ΦOTDR (CP-ΦOTDR) technology represents an advancement that overcomes the aforementioned limitations present in conventional ΦOTDR. By implementing a linear variation in the emission frequency within the pulse, the CP-ΦOTDR system effectively addresses strain-induced variations in the refractive index. The technique is rooted in the estimation of local temporal delay (Δ*t*) resulting from a perturbation-induced spectral shift (Δν) [23]. This Δ*t* results from group refractive index variations ($\Delta n_{g}$) and correlates directly with ongoing strain perturbations [24]. Equation (1) elucidates how strain variations, Δε, are derived from local temporal shifts in the trace, Δ*t* [23,25].

(1) $$-\frac{1}{\nu_{0}}\frac{\delta \nu_{p}}{\tau_{p}}\Delta t=\frac{\Delta n_{g}}{n_{g}}\approx \kappa_{\varepsilon }\cdot \Delta \varepsilon$$

where $\nu_{0}$ stands for the central frequency of the pulse, $n_{g}$ corresponds to the group refractive index, $\tau_{p}$ stands for the temporal width of the pulse, $\delta \nu_{p}$ stands for the spectral content of the linearly chirped pulse, and $\kappa_{\varepsilon }$ stands for a constant with value −0.78. Significantly, the measurement principle in CP-ΦOTDR is not directly tied to the intensity of the backscattered trace, leading to a Signal-to-Noise Ratio (SNR) that does not exhibit exponential decay with the sensing distance, as observed in conventional ΦOTDR.

## 3. Data and Measurements

**Figure 1:**
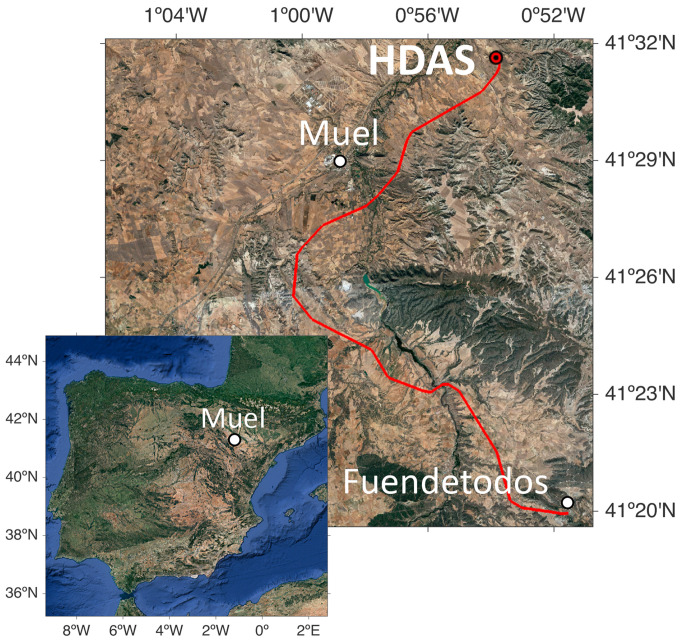
Location of the experimental overhead line installation, shown in red.

**Figure 2:**
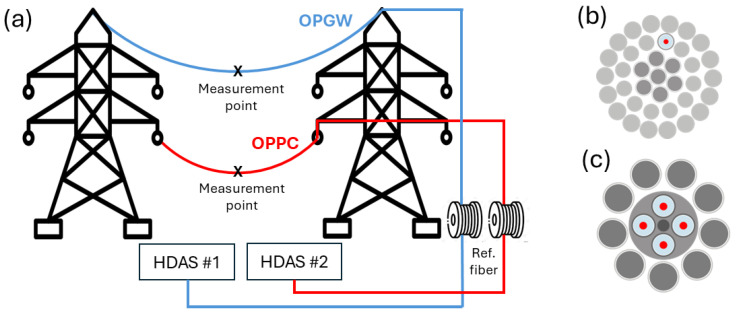
(**a**) Overhead installation scheme. (**b**) OPPC (fiber in red). (**c**) OPGW (fiber in red).

The measurements for this study were conducted in February 2022 on the María–Fuendetodos experimental overhead line (Figure 1). The line, operated by Red Eléctrica de España, is located in northeast Spain and is mainly used to evacuate the energy generated by a nearby wind turbine installation. The overhead line consists of two double-circuit 220 kV lines and both an Optical Phase Conductor (OPPC) and Optical Ground Wire (OPGW), containing several standard communication optical fibers (G652) deployed as shown in Figure 2b,c. The OPGW has a thick inner aluminium core that protects the optical fibers in its interior and provides it with more rigidity and stability, while the OPPC has the fibers braided like the rest of its threads, without any additional protective core, allowing for greater flexibility on the conductor.

Both of these cables were monitored in this study. To do so, a commercial High-Fidelity Distributed Acoustic Sensor (HDAS) was provided and installed by Aragón Photonics Labs in the substation located in María de Huerva, Spain. This interrogator unit was set with a peak power output of 200 mW, a 100 ns pulse duration, and a linear chirp over the pulse emission. The HDAS was calibrated to a 1 nϵ strain resolution and a sensing distance of 50 km, with a 10 m spatial resolution and an effective acoustic sampling rate of 250 Hz. In order to compensate for the laser phase noise contribution, a 1.2 km launching fiber was placed between the interrogator and the connector to both the OPGW and OPPC. By keeping this fiber unperturbed in an isolated foam box inside the station, the laser phase noise can be estimated and compensated for over the entire sensing distance, with up to 17 dB of improvement in the SNR [26]. The full scheme of the experimental setup is shown in Figure 2a.

**Figure 3:**
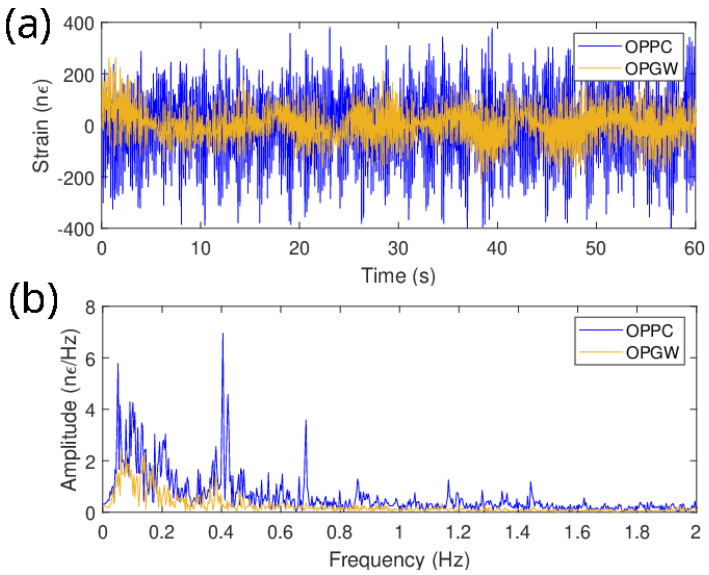
One-minute data record of CH552 for the OPPC (blue) and the OPGW (yellow). (**a**) Strain. (**b**) Frequency spectrum.

Figure 3a illustrates the strain for both the OPPC and OPGW of the central point (CH552) in a 317 m long span with a northeast transversal direction, located approximately 5.5 km away from the interrogator, during a 1 min record at 07:00 local time. Prior to any analysis, a 20 s filter was applied to eliminate low-frequency noise resulting from extended integration times. It is clearly evident from the data that, under similar external stimuli, the OPPC undergoes significantly greater deformation than the OPGW, consistent with their internal structures and reflected by the higher strain values shown in Figure 3a. The strain data spectrum for both cables is depicted in Figure 3b. This representation reveals distinctive frequencies with elevated amplitudes for both cables, intrinsic to the mechanical and structural properties of each span and wire. These characteristic frequencies are directly correlated with the instantaneous sag value and also reflect the mechanical condition of the cable, indicating whether it is stable or under perturbation.

## 4. High-Frequency Range: On-Field Intervention Monitoring

**Figure 4:**
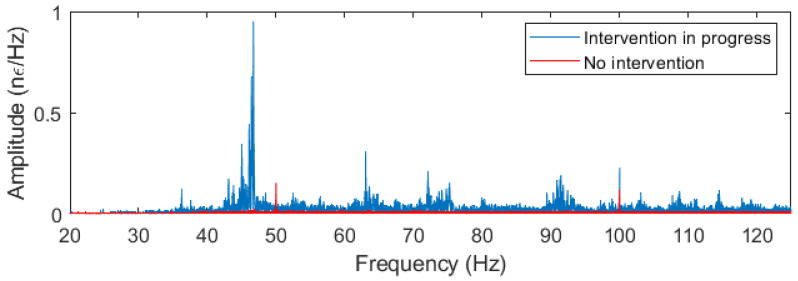
Frequency data of the OPPC with (blue) and without (red) mechanical intervention on the tower.

By analyzing the high-frequency component of the measured data (typically above 20 Hz), it is possible to monitor a wide range of mechanical interventions on overhead line structures. Routine maintenance activities, such as tower inspections and repairs, as well as more disruptive events, such as cable failures or unauthorized access, can be readily identified in the frequency spectrum of the measured data. Figure 4 illustrates a clear distinction between the high-frequency data collected during intervention periods and the baseline data during non-intervention periods. The data represented in the figure correspond to the frequency components of the strain measured over 1 s intervals at the location of kilometer 25, where maintenance was occurring on the nearest suspension tower.

**Figure 5:**
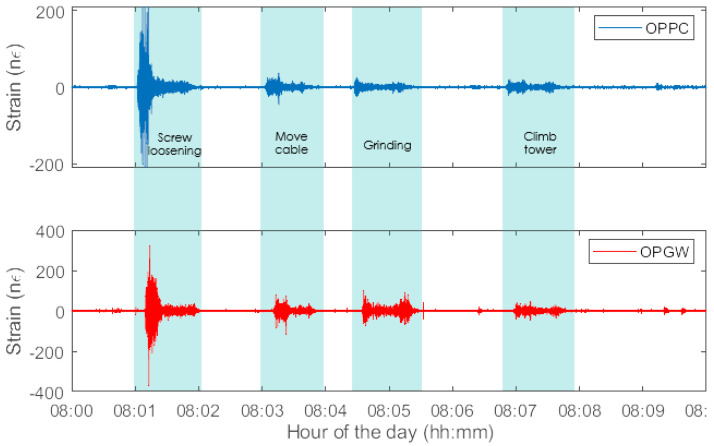
Measured strain during interventions on both the OPPC (blue) and the OPGW (red).

A series of line maintenance operations were conducted on a suspension tower while both the OPPC and OPGW were continuously monitored. These interventions included actions such as loosening of fastener screws, material removal using a grinding tool, and personnel movements on the tower structure, including climbing up and down the tower. The impact of these activities on the mechanical strain of both cables is clearly discernible in the measured strain presented in Figure 5, which shows distinct patterns corresponding to each activity. This analysis supports the reliable identification of specific maintenance tasks based on frequency characteristics.

**Figure 6:**
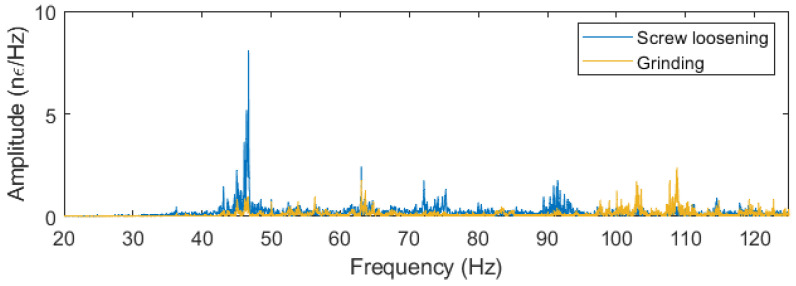
Frequency spectrum of the screw loosening intervention (blue) and the material grinding (yellow).

The inherent linearity of the measurement technique enables the discrimination between the various interventions through the identification of their respective characteristic frequencies. Each type of intervention will present a unique frequency spectrum, which can be studied and classified in order to automatically detect what is currently happening in the overhead line installation. For example, Figure 6 demonstrates the frequency spectrum differences between screw loosening (blue) and grinding (yellow). These distinct spectral characteristics enable an automated classification system, enhancing the monitoring system’s ability to distinguish among various on-field activities and assess their impact on overhead line structures.

## 5. Low-Frequency Range: Sag Estimation

The determination of the sag in a suspended cable can be directly derived from the analysis of its low-frequency vibrational data [27]. In a simplified model, where suspension towers are considered rigid, the overhead conductor can be represented as a cable with both ends fixed and no stiffness. This simplification establishes a direct link between the fundamental vibration frequency of each span and the corresponding span length and tension. Equation (2) encapsulates the correlation between the sag *S*, the length of the conductor *L*, the weight mass per length unit $m_{c}$, and the cable tension *H*, where *g* corresponds to the gravity constant and is equal to 9.81 m/s^2^.

(2) $$S=\frac{gL^{2}m_{c}}{8H}$$

All the main parameters of the conductor have a unique contribution to the fundamental frequency $f_{0}$ of each span, as depicted in Equation (3). This fact leads to a direct relationship between this fundamental frequency and the sag of the conductor in the analyzed span.

(3) $$H=4m_{c}{\left(Lf_{0}\right)}^{2}$$

Hence, the determination of sag can be directly achieved through the fundamental vibration frequency, and it remains impervious to external influences. This inherent relationship allows for an instantaneous and accurate estimation of sag for each span by an in-depth frequency analysis. In addition, this process eliminates the necessity for amplitude calibration, streamlining the sag assessment by relying solely on the dynamic characteristics fully encapsulated in the fundamental frequency data.

As no substantial alterations were observed in the mass of the monitored lines, the main variable in Equation (2) is their length. Environmental temperature fluctuations induce elongation in both the OPPC and the OPGW. While the OPPC may exhibit more pronounced sag variations due to the Joule effect resulting from conduction, it is important to note that the line remained inactive during the monitoring period.

The elongation caused by temperature changes leads to variations in tension, and both these alterations are uniquely reflected in the fundamental frequency of vibration. For a span of 400 m, typical sag values hover around 10 m, resulting in fundamental frequencies approximately at 0.17 Hz [28].

**Figure 7:**
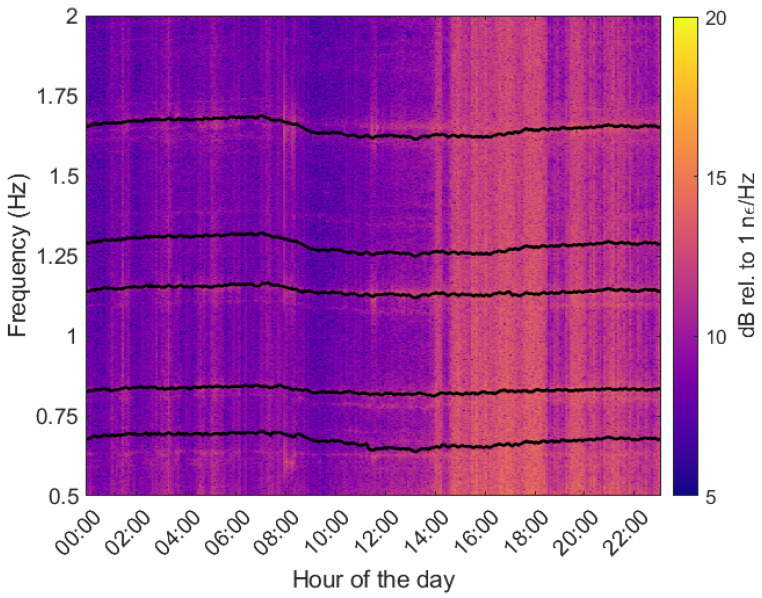
Some of the frequency lines detected by the algorithm in the 24 h monitored spectrogram of CH552.

**Figure 8:**
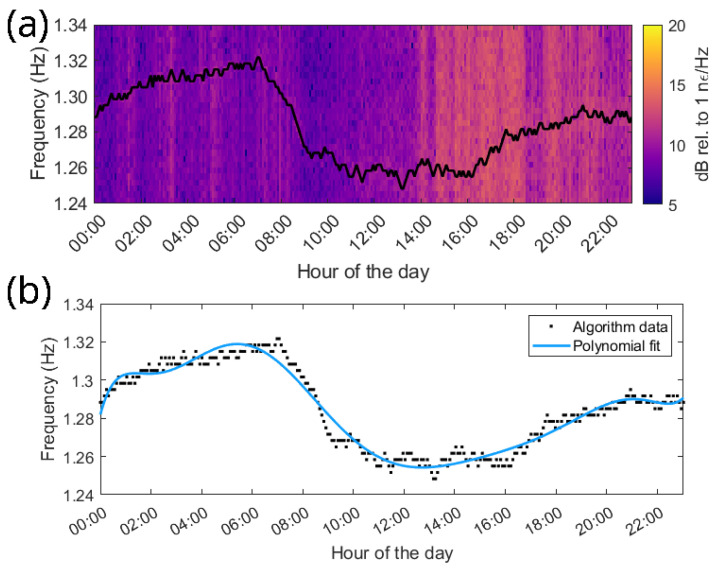
(**a**) Evolution of the 8th harmonic of CH552 (OPPC). (**b**) Peak-tracking algorithm data and polynomial fit of the harmonic mode.

A previous study showed that analyzing the spectrogram of a complete 24 h duration allows for the observation of the distinctive vibration frequencies exhibited by the conductor within a specific span [11]. These frequencies manifest as prominent lines, undergoing variations throughout the measured period. The dynamic evolution of these detected frequencies in the OPPC is graphically depicted in Figure 7. Notably, the spectrogram reveals the presence of several harmonic vibrational modes. To track the progression of these frequencies over the 24 h measurement period, a peak-tracking algorithm was employed. The resulting analysis reveals that the frequency attains its maximum value around 08:00H and reaches a minimum around 14:00H, as shown in Figure 8a. These temporal extremes align with the recorded minimum and maximum environmental temperatures at a meteorological station situated around 12 km from the power line. The station is placed in Valmadrid; it is operated by the State Meteorological Agency (AEMET) and provides publicly accessible data.

Figure 8b illustrates the result of the peak-tracking algorithm dynamic changes in frequency for one of the harmonics identified in the OPPC’s spectrogram. This depiction includes a polynomial adjustment along with the recorded temperature variations throughout the 24 h measurement period. Considering the linear increase in sag with the temperature, as outlined by Equation (2), an inherent inverse correlation between frequency and temperature is expected.

**Figure 9:**
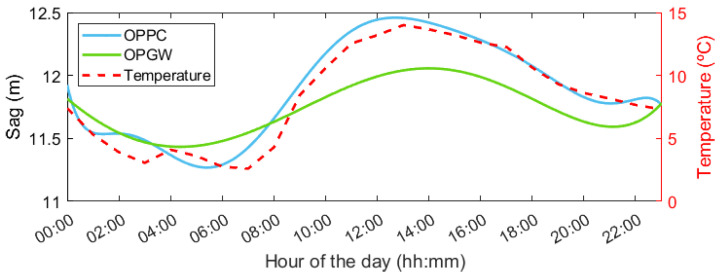
Sag evolution in the 24 h period for CH552 obtained using CP-ΦOTDR both in the OPPC and OPGW.

Based on the featured frequencies observed in Figure 7 for the OPPC, a fundamental frequency of 0.168 Hz can be deduced. This frequency exhibits temporal fluctuations with variations extending up to 0.01 Hz. By using Equation (2), it becomes possible to track the absolute sag values across the monitored span throughout a 24 h duration. The same analytical approach has been extended to the OPGW, yielding the final sag values depicted in Figure 9. The OPGW experienced a maximum sag differential of 0.62 m, while the OPPC recorded a more significant sag variation, reaching up to 1.19 m. During the monitored 24 h period, no electrical conduction took place, implying that the elongation of both cables was primarily influenced by environmental temperature variations. Since both cables experience the same daily temperature fluctuations, the temporal pattern of the sag values depicted in Figure 9 is correspondingly similar, differing only in the amplitude values. It is noteworthy that the OPGW features an internal aluminum core designed to safeguard optical fibers, enhancing the cable’s rigidity. Consequently, this structural distinction results in lower sag values for the OPGW compared to the OPPC [29]. The increased flexibility of the OPPC, stemming from its design, allows for greater elongation, directly manifesting in more pronounced frequency variations in the harmonic modes identified in the spectrogram.

## 6. Conclusions

In this study, CP-ΦOTDR technology was employed to monitor both the OPPC and OPGW optical fibers along a 31.8 km overhead line, analyzing the vibrations induced on both cables. The inherent capabilities of this DAS technology facilitate the precise frequency analysis of diverse events taking place within the overhead line installation. High-frequency data filtering enabled the detection and classification of various mechanical interventions on suspension towers. In the low-frequency range of the spectrum, spectrograms were computed, revealing distinct featured frequencies associated with vibration phenomena. These frequencies displayed a periodic variation highly correlated with the ambient temperature fluctuations. Significantly, these featured frequencies were linked to the sag of the conductor within the measured span, and their variations were directly related with the thermal elongation or contraction of the conductor. The identification of the fundamental vibrational frequency enabled the calculation of instantaneous sag values for both cables. The estimated sag values over a 24 h period exhibited discernible differences between the studied cables due to their structural differences, yet the sag evolution of both overhead cables remarkably aligned with the recorded environmental temperature variations. Simultaneous measurement of the conductor (OPPC) and the ground line (OPGW) enables precise monitoring of the conductor’s sag. Information from the ground line helps estimate the part of elongation attributable to environmental temperature changes, providing a more accurate assessment of the OPPC’s condition in response to electrical conduction. The method presented in this study demonstrates its effectiveness in estimating instantaneous sag through vibrational frequency analysis obtained with CP-ΦOTDR, offering a cost-efficient solution to sag monitoring with strong potential for real-world application. Additionally, the system enables real-time threat monitoring by identifying and classifying various mechanical interventions and disturbances on the line. This dual functionality not only supports proactive maintenance and operational efficiency but also enhances infrastructure security by allowing for immediate detection of unauthorized or potentially damaging activities along the transmission line. The current study demonstrates the feasibility of a DAS-based monitoring system using a straightforward FFT analysis approach. To further enhance the system’s capabilities and robustness, particularly in challenging noise conditions, the incorporation of advanced signal processing techniques, such as the activation function dynamic averaging method [30] or similar nonlinear methods, could be explored. These techniques have the potential to improve frequency resolution and reduce the impact of noise, thereby expanding the system’s operational range and leading to a more comprehensive and robust monitoring solution.

## Data Availability

The data presented in this study are available on request from the corresponding author due to privacy reasons.

## Abbreviations

The following abbreviations are used in this manuscript:

DAS	Distributed Acoustic Sensing
DFOS	Distributed Fiber Optic Sensing
CP-ΦOTDR	Chirped-Pulse Phase-Sensitive Optical Time-Domain Reflectometry
OPGW	Optical Ground Wire
OPPC	Optical Phase Conductor
ADSS	All Dielectric Self Supported
I/Q	In phase and Quadrature
SNR	Signal-to-Noise Ratio
HDAS	High-Fidelity Distributed Acoustic Sensor
AEMET	Agencia Estatal de Meteorología
